# Quercetin Inhibits IL-1β-Induced Inflammation, Hyaluronan Production and Adipogenesis in Orbital Fibroblasts from Graves' Orbitopathy

**DOI:** 10.1371/journal.pone.0026261

**Published:** 2011-10-19

**Authors:** Jin Sook Yoon, Hyun Jung Lee, Soo Hyun Choi, Eun-Ju Chang, Sang Yeul Lee, Eun Jig Lee

**Affiliations:** 1 Institute of Vision Research, Department of Ophthalmology, Yonsei University College of Medicine, Seoul, Korea; 2 Endocrinology, Brain Korea 21 Project for Medical Science, Institute of Endocrine Research, and Severance Integrative Research Institute for Cerebral & Cardiovascular Disease, Seoul, Korea; 3 Department of Anatomy and Cell Biology, Cellular Dysfunction Research Center, University of Ulsan College of Medicine, Seoul, Korea; 4 Biochemistry and Molecular Biology, Yonsei University College of Medicine, Seoul, Korea; University of Muenster, Germany

## Abstract

Management of Graves' orbitopathy (GO) is challenging, as no reliable, specific, and safe medical therapeutic agents have yet been developed. We investigated the effect of quercetin in primary cultured orbital fibroblasts from GO, targeting pathways of inflammation, aberrant accumulation of extracellular matrix macromolecules, and adipose tissue expansion. Quercetin significantly attenuated intercellular adhesion molecule-1 (ICAM-1), interleukin (IL) -6, IL-8, and cyclooxygenase (COX) -2 mRNA expression, and inhibited IL-1β-induced increases in ICAM-1, IL-6, and IL-8 mRNA. Increased hyaluronan production induced by IL-1β or tumor necrosis factor-α was suppressed by quercetin in a dose- and time-dependent manner. Treatment with noncytotoxic doses of quercetin inhibited accumulation of intracytoplasmic lipid droplets and resulted in a dose-dependent decrease in expression of peroxisome proliferator-activated receptor γ, CCAAT/enhancer-binding protein (C/EBP) α, and C/EBPβ proteins. In conclusion, inhibition of inflammation, hyaluronan production, and adipogenesis by the natural plant product quercetin in vitro provides the basis for further study of its potential use in the treatment of GO.

## Introduction

Graves' disease is an autoimmune disease of the thyroid gland in which autoantibodies bind to the thyrotropin receptor on thyroid follicular cells, thereby activating gland function and leading to excess production of thyroid hormones. Up to 50% of Graves' disease patients develop manifestations pathologic in the eye, known as Graves' orbitopathy (GO) [1], [2]. The most common features of GO include upper eyelid retraction, edema, erythema of periorbital tissues, and proptosis. Between 3–5% of individuals with GO suffer from intense pain and inflammation, diplopia, and sight-threatening compressive optic neuropathy.

An increase in connective/fatty tissues within the bony orbits is responsible for most orbital complications in patients with severe active GO [3]. Tissue expansion is characterized by marked infiltration of immunocompetent cells, mainly T and B lymphocytes and mast cells, and the presence of abundant hydrophilic glycosaminoglycans, predominantly hyaluronan. It is likely that orbital adipose tissue in GO is more cellular than normal and contains a higher proportion of preadipocytes capable of differentiating into adipocytes [4], [5].

GO is a disfiguring and often incapacitating disease that is difficult to treat. Glucocorticoids have been used for decades and are still indicated as the first-line treatment because of their anti-inflammatory and immunosuppressive actions, either alone or in combination with orbital radiotherapy [2], [6]. Glucocorticoids are mostly effective in patients with severe and active eye disease [6]. However, proptosis and longstanding extraocular muscle involvement associated with fibrotic changes are poorly responsive. Another drawback of glucocorticoid therapy is the long-term side effects, including cushingoid features, diabetes, hypertension, osteoporosis. No reliable, specific, and safe medical therapeutic agents have yet been developed for GO. The development of specific therapies targeting pathways of inflammation, adipose tissue expansion, aberrant accumulation of extracellular matrix macromolecules, and fibrosis is essential.

Quercetin (3, 3, 4, 5, 7-pentahydroxy flavonone) is a flavonoid phytoestrogen, found abundantly in soybeans, vegetables, and fruits. Quercetin affects cell cycle kinetics and proliferation and induces apoptosis [7], [8]. Quercetin has also been found to possess antioxidant [9], anti-inflammatory [10], [11], and antiadipogenic properties [12], [13], [14]. Recently, Lisi et al. reported that quercetin reduced cell proliferation and hyaluronan release in orbital fibroblasts when cells were incubated for 3–5 days, and the mechanism responsible for inhibition of cell proliferation was the induction of necrosis as well as cell cycle blockade [15].

In this study, we chose to employ noncytotoxic conditions of quercetin exposure to investigate its inhibitory effects on inflammation, hyaluronan production, and adipogenesis, thereby targeting the three major mechanistic pathways of GO.

## Results

### Effect of quercetin on the viability of orbital fibroblasts

**Figure 1:**
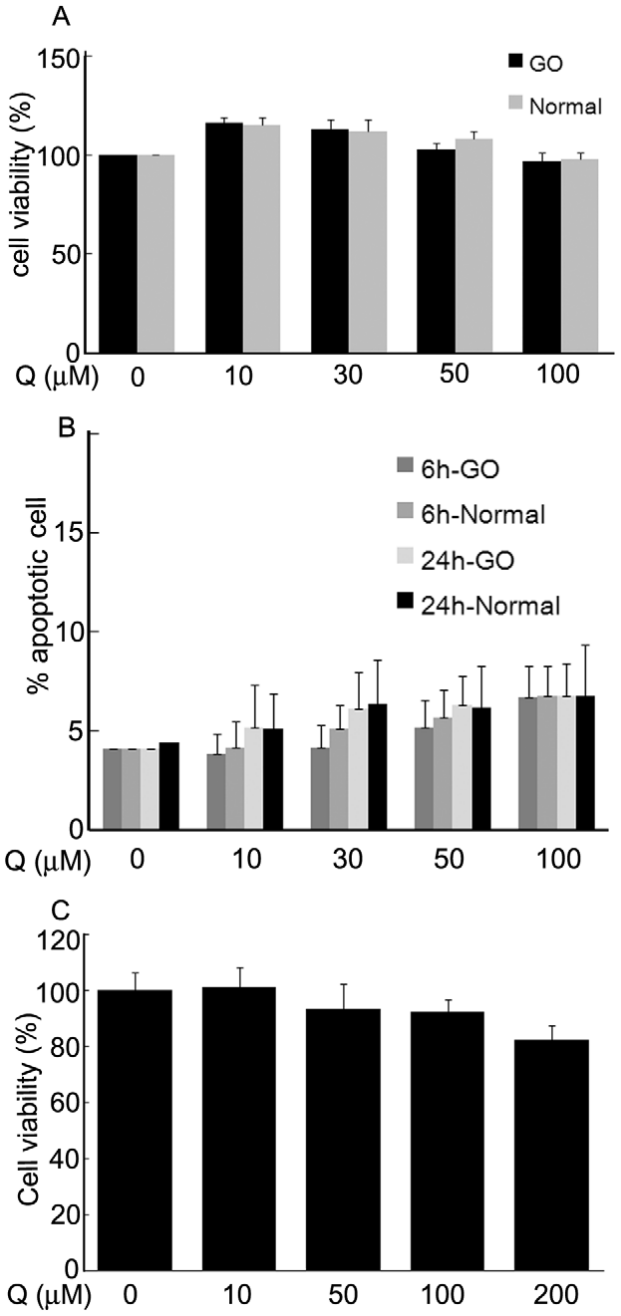
Effect of quercetin on cell viability and apoptosis in preadipocyte orbital fibroblasts and differentiating orbital fibroblasts. (A) Orbital fibroblasts (1×10^5^) of normal and Graves' orbitopathy (GO) patients were seeded into 24-well culture plates and treated with different concentrations of quercetin (10, 30, 50, or 100 µM) for 24 h. After treatment, assays with 3-(4, 5-dimethyl-thiazol-2-yl)-2, 5-diphenyl-tetrazolium bromide (MTT) were performed to test for viability. (B) An annexin V/FITC kit was used to detect phosphatidylserine externalization, as an index of apoptosis. Percentage of stained cells with annexin V was analyzed by flow cytometry. (C) Orbital fibroblasts (1×10^5^) of GO patients were seeded into 24-well culture plates and treated with different concentrations of quercetin (10, 50, 100, or 200 µM) for 3 days in adipogenic medium containing adipogenesis inducers and rosiglitazone (10 µM). After treatment, MTT assays were performed. Results are expressed as percentage of untreated control values presented as mean ± standard deviation (SD). Assays were performed at least three times in triplicate; data from a representative experiment are shown, expressed as the differences between treated and untreated cells.

To determine nontoxic, concentrations of quercetin in orbital fibroblasts, 3-(4,5-dimethyl-thiazol-2-yl)-2,5-diphenyl-tetrazolium bromide (MTT) assay and annexin V-fluorescence isothiocyanate (FITC) apoptosis assay were performed. Exposure of cells to quercetin at ≤100 µM for 24 h neither decreased cell viability below 95% in both normal and GO orbital fibroblasts (Fig. 1A) nor induced a significant level of apoptosis (less than 7%; Fig. 1B). Therefore, treatment of cells with 100 µM quercetin for 24 h was used to determine effects on inflammation and hyaluronan production. For experiments testing suppression of adipogenesis, cells in adipogenic medium were treated with quercetin (10–200 µM) from days 1–3 of differentiation. Cell viability at day three in the presence of 100 µM quercetin was not significantly reduced compared to untreated cells, whereas 200 µM attenuated cell viability to 82.3% (Fig. 1C). Therefore noncytotoxic concentrations (50, 100 µM) of quercetin were used for 3 days in differentiating cells cultures to find the effect of quercetin on adipogenesis.

### Effect of quercetin on the expression of mRNA of interleukin (IL) -1β-induced proinflammatory molecules

**Figure 2:**
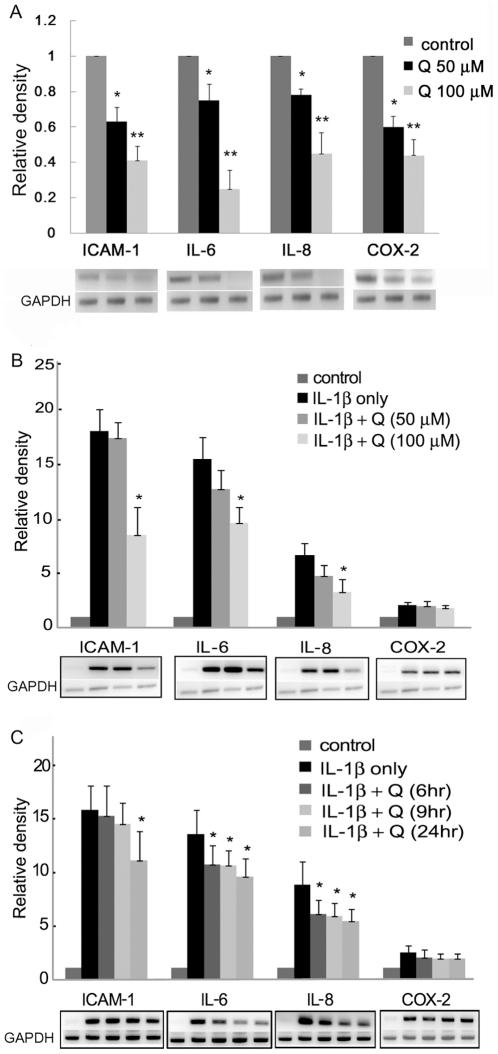
Effects of quercetin on ICAM-1, IL-6, IL-8, and COX-2 mRNA expression in Graves' orbitopathy (GO) orbital fibroblasts. (A) Orbital fibroblasts (5×10^5^) from GO patients pretreated with 0, 50, or 100 µM quercetin for 24 h were used to analyze for ICAM-1, IL-6, IL-8, and COX-2 mRNA expression by RT-PCR. (B) Cells pretreated as in (A) were then stimulated with IL-1β (10 ng/ml) for 16 h, and were then used for RT-PCR analyses. (C) RT-PCR analysis of ICAM-1, IL-6, IL-8, and COX-2 mRNA expression, with values determined by densitometry and normalized to GAPDH. Cells had been pretreated with 100 µM quercetin for 6, 9, or 24 h, then stimulated with IL-1β (10 ng/ml) for 16 h. Data in each column above represent the mean relative density ratio ± SD of three experiments, and representative gel images are shown below the graphs. Differences between treated and untreated cells (*P<0.05, **P<0.001) are indicated.

We first examined intercellular adhesion molecule (ICAM) -1, IL-6, IL-8 and cyclooxygenase (COX)-2 gene expression in the absence or presence of quercetin (50 or 100 µM for 24 h), evaluated in both GO and normal orbital fibroblasts by reverse transcription-polymerase chain reaction (RT-PCR). These proinflammatory molecules were virtually undetectable in untreated normal cell cultures (data not shown) but were detectable in GO cells, and expression was decreased significantly by quercetin pretreatment in a dose-dependent manner (Fig. 2A).

Then we tested for stimulation of ICAM-1, IL-6, IL-8, and COX-2 gene expression in both normal and GO cells by IL-1β (10 ng/ml for 16 h), with or without quercetin pretreatment. Expression of ICAM-1, IL-6, IL-8, and COX-2 mRNA was strongly upregulated by IL-1β in both GO and normal cells, and there was no difference observed between GO and normal cells (data not shown). Quercetin suppressed IL-1β-stimulated ICAM-1, IL-6, and IL-8 mRNA similarly in both GO and normal cells in a dose- and time-dependent manner (*P*<0.05). Fig. 2B shows the dose-dependent suppressive effects of quercetin in GO orbital fibroblasts (normal data not shown; Fig. 2B). The longer pretreatment with quercetin resulted in greater suppression of the IL-1β-induced upregulation of these three proinflammatory molecules (Fig. 2C). In contrast, the IL-1β-induced COX-2 mRNA level was not significantly altered by quercetin pretreatment.

To investigate the effect of quercetin on IL-10 production, we examined IL-10 mRNA expression in GO cells cultured with quercetin in various concentrations (0–100 µM) (Fig. S1A). IL-10 mRNA expression was very weak in control cells, and was not affected by the quercetin treatment. IL-1β (10 ng/ml) did not induce IL-10 expression, and the quercetin did not change its level (Fig. S1 B).

### Effect of quercetin on IL-1β- or tumor necrosis factor (TNF)-α-induced hyaluronan production

**Figure 3:**
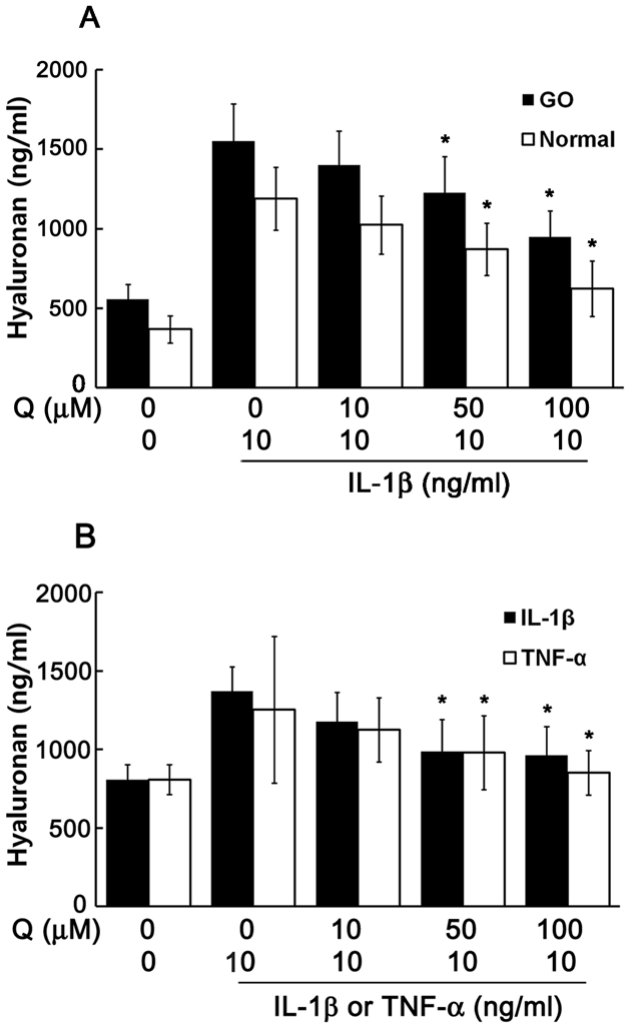
The effect of quercetin on hyaluronan production induced by IL-1β or TNF-α in orbital fibroblasts. (A) Hyaluronan in media of confluent orbital fibroblast cultures from GO (n = 3) and normal (n = 3) individuals pretreated with 0, 10, 50 or 100 µM quercetin for 24 h before IL-1β stimulation (10 ng/ml, 16 h). (B) The effect of quercetin on hyaluronan production in GO orbital fibroblasts (n = 3) stimulated with IL-1β (10 ng/ml, 16 h) or TNF-α (10 ng/ml, 16 h). Triplicate measurements were averaged, and the data are expressed as mean values ± SD. *P<0.05 vs. cells stimulated with IL-1β or TNF-α alone.

Hyaluronan concentrations in culture medium did not differ significantly between unstimulated normal (367±85 ng/ml) and GO orbital fibroblasts (557±94 ng/ml; *P* = 0.07). However, the IL-1β-stimulated hyaluronan concentration was significantly higher in GO (1552±234 ng/ml) than in normal cultures (1191±198 ng/ml; *P* = 0.023). Quercetin pretreatment (50 and 100 µM) significantly reduced the IL-1β-induced hyaluronan release in both GO (1226 and 947 ng/ml, respectively) and normal cells (871 and 624 ng/ml, respectively; *P*<0.05; Fig. 3A). The effect of quercetin pretreatment on hyaluronan production induced by either IL-1β or TNF-α in GO orbital fibroblasts is shown in Fig. 3B. TNF-α (10 ng/ml) stimulated hyaluronan production to levels similar to those induced by IL-1β, and quercetin pretreatment significantly lowered this production in a dose-dependent manner (all *P*<0.05).

### Suppressive effect of quercetin on nuclear factor (NF)-κB activation

**Figure 4:**
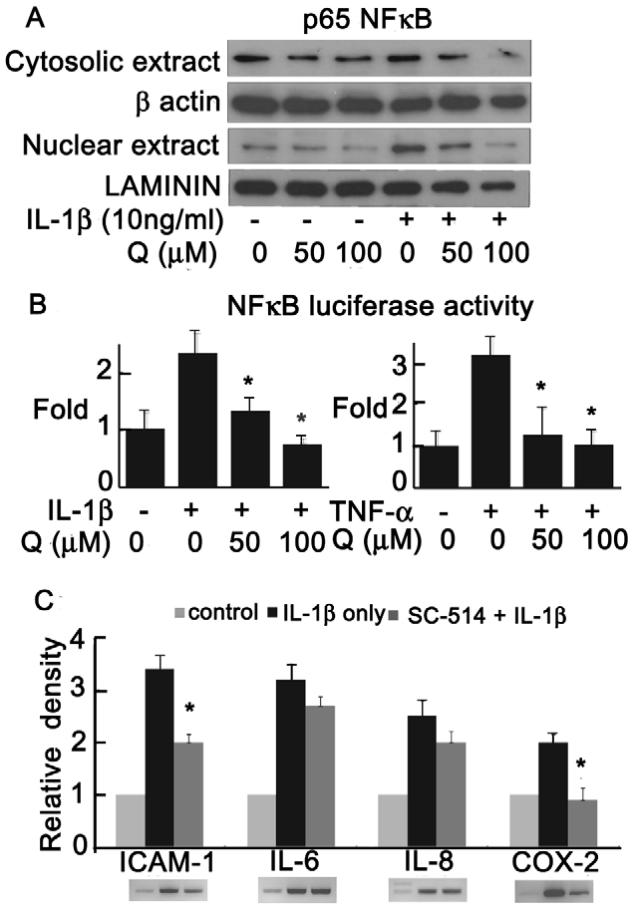
Effect of quercetin on NF-κB activation in GO orbital fibroblasts. (A) Cells were pretreated with quercetin for 24 h prior to IL-1β (10 ng/ml) stimulation for 16 h, and p65 NF-κB translocation was assayed by western blot analysis. (B) Results of assays measuring NF-κB activity with a NF-κB-dependent luciferase reporter construct in cells treated with quercetin (0, 50 or 100 µM, 24 h) prior to stimulation with IL-1β or TNF-α (10 ng/ml) for 16 h. (C) RT-PCR analysis of ICAM-1, IL-6, IL-8, and COX-2 expression in cells pretreated with NF-κB inhibitor SC-514 (100 µM) for 1 h and then stimulated with IL-1β (10 ng/ml). PCR bands measured by densitometry and normalized to GAPDH. *P<0.05 vs. cells stimulated with IL-1β or TNF-α alone.

Because the NF-κB signaling pathway regulates the production of many cytokines, we investigated effects of IL-1β and quercetin on the nuclear translocation of active NF-κB in orbital fibroblasts from GO patients. As shown in Fig. 4A, stimulation of orbital fibroblasts with IL-1β induced nuclear translocation of p65 NF-κB, and quercetin dose-dependently inhibited this. We examined the effect of quercetin using an NF-κB-dependent luciferase reporter assay. Quercetin significantly reduced the IL-1β- or TNF-α-induced elevation of luciferase activity in a dose-dependent manner (Fig. 4B). To determine whether the IL-1β-stimulation of proinflammatory gene expression was mediated by an NF-κB-dependent pathway, SC-514, a selective IκB kinase-2 inhibitor, was tested. We found that preincubation with SC-514 (100 µM) for 1 h significantly decreased IL-1β-induced ICAM-1 and COX-2 gene expression, but the decreases in IL-6 and IL-8 mRNA were not significant (Fig. 4C), suggesting the presence of different activation mechanisms for these proinflammatory genes.

### Effect of quercetin on adipogenesis in GO orbital fibroblasts

Confluent orbital fibroblasts from GO patients were subjected to an adipocyte differentiation protocol for 10 days and examined by light microscopy. Under the control adipogenic conditions without rosiglitazone, orbital fibroblasts lost their stellate fibroblastic appearance and converted to a spherical adipocytic shape, and a fraction of these cells accumulated small lipid droplets. Visible from day 3, the lipid droplets increased in number and enlarged in size during the 10 days of differentiation (Fig. S2 A). The addition of rosiglitazone (10 µM) significantly increased adipogenesis (Fig. S2 B). Treatment with IL-1β (10 ng/ml) increased cellular accumulation of lipid droplets (Fig. S2 C), as reported previously [16]. Combined treatment with rosiglitazone and IL-1β further stimulated adipogenesis (Fig. S2 D).

**Figure 5:**
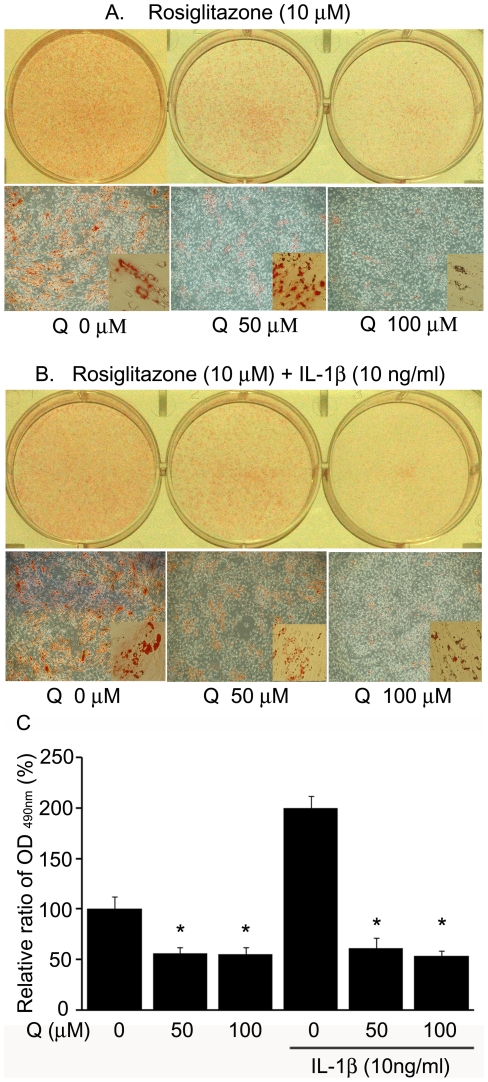
Effect of quercetin on adipogenesis in GO orbital fibroblasts. (A–B) Quercetin (50 or 100 µM) treatment for the first 3 days after initiation of 10-day adipogenesis in adipogenic media containing (A) 10 µM rosiglitazone, or (B) combined 10 µM rosiglitazone and 10 ng/ml IL-1β. Cells were stained with Oil Red O and examined grossly and microscopically (×40; inset ×400). (C) Cell-bound Oil Red O was solubilized and optical density (OD) read at 490 nm to obtain a quantitative assessment of adipogenesis. The experiments were performed in triplicate with cells from three different donors, and data in the column are the mean relative density ratios ± SD of three experiments. *P<0.001 vs. untreated control differentiated cells.

Oil Red O staining showed that quercetin dose-dependently decreased the size and number of intracytoplasmic lipid droplets in cells treated with either rosiglitazone alone or in combination with IL-1β (Fig. 5A and B). The optical density of stained cell lysates was measured to evaluate adipocyte differentiation quantitatively (Fig. 5C). Quercetin-treated cells showed significantly decreased absorbance at 490 nm in a dose-dependent manner (*P*<0.001). IL-1β treatment stimulated higher lipid accumulation levels, and the stimulatory effect was inhibited by quercetin (*P*<0.001).

### Effect of quercetin on the expression of transcriptional regulators of adipogenesis

**Figure 6:**
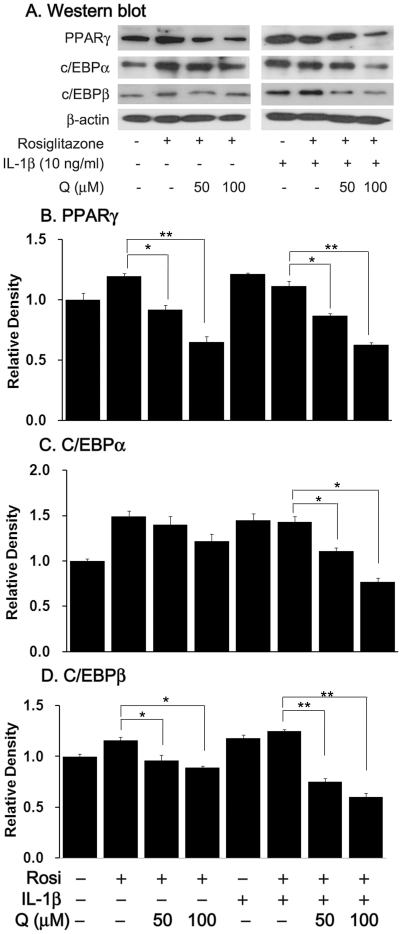
Effect of quercetin on the expression of adipogenic transcriptional regulators in differentiated orbital fibroblasts from GO patients. (A) Quercetin (50 or 100 µM) treatment for the first 3 days after initiation of 10-day adipogenesis in adipogenic media containing 10 µM rosiglitazone, or combined 10 µM rosiglitazone and 10 ng/ml IL-1β. After 10 days, cell lysates were subjected to western blot analysis of PPARγ, C/EBPα, and C/EBPβ protein expression. The experiments were performed in triplicate with cells from three different donors. (B–D) Quantification by densitometry, normalized to the β-actin level in the same sample, is shown for PPARγ (B), C/EBPα (C), and C/EBPβ (D). The data in the column are the mean relative density ratios ± SD of three experiments. *P<0.05, **P<0.001 vs. untreated control differentiated cells.

Western blot analysis was performed to investigate whether quercetin affects the expression of adipogenic transcription factors. As shown in Fig. 6, peroxisome proliferator-activated receptor (PPAR) γ and CCAAT/enhancer-binding proteins (C/EBP) α and β were all strongly enhanced in cells treated with either rosiglitazone or IL-1β. The protein levels were all further increased by the combination of rosiglitazone and IL-1β. Quercetin dose-dependently and significantly attenuated the expression of PPARγ, C/EBPα, and C/EBPβ in differentiated fibroblasts treated with rosiglitazone, with or without IL-1β (Fig. 6A, B, C, and D).

## Discussion

In this study, we found that quercetin blocks three biological processes—inflammation, aberrant accumulation of extracellular matrix macromolecules, and adipose tissue expansion—in primary cultured orbital fibroblasts from GO stimulated by proinflammatory cytokines. Significantly, these three processes are the major pathogenic mechanisms associated with development of GO.

In the early stage of GO, infiltrating T cells interact with orbital fibroblasts, potentially resulting in cross-activation, further promoting cytokine production and secretion of T-cell activating factors by fibroblasts, such as IL-8 and products of COX-2 activity [3]. Many observations support this hypothesis. For example, stimulated fibroblasts secrete multiple cytokines, including IL-6, which stimulates B cell differentiation and thus Graves' disease IgG [17]. ICAM-1 mRNA and protein are upregulated in GO orbital fibroblasts by CD40 ligand, mainly through the NF-κB and p38 pathways [18]. IL-1α, TNF-α, and interferon-γ stimulate the expression of ICAM-1 in GO orbital fibroblasts [19]. IL-1β increases production of IL-6 and IL-8 in orbital fibroblasts [20], [21], [22], [23]. Additionally, active GO tissues have higher levels of mRNA for IL-6 and IL-8 than inactive GO tissues [24]. In our study, we found that quercetin inhibits ICAM-1, IL-6, IL-8, and COX-2 gene expression in cell strains from GO donors, and it also suppresses IL-1β-induced ICAM-1, IL-6, and IL-8 mRNA expression in cell strains from GO and normal donors. Considering the possible roles of ICAM-1, IL-6, IL-8, and COX-2 in the immunologic pathogenesis of GO, prevention of expression of these molecules could be an effective treatment for GO.

There are conflicting reports concerning the expression of COX-1 and -2 in orbital fibroblasts [25], [26]. In our study, we observed that COX-2 mRNA expression is significantly inhibited by quercetin treatment in GO cell strains. We found that COX-2 mRNA is upregulated by IL-1β in both GO and normal cells; however, quercetin did not suppress the IL-1β-induced increase of COX-2 gene expression. Of note, the relative expression of COX-2 mRNA stimulated by IL-1β was lower than that of ICAM-1, IL-6, and IL-8 in all cell cultures.

We expected to observe a more significant upregulation of proinflammatory molecule gene expression by IL-1β or TNF-α in GO cell strains than in normal cells, but we obtained similar results in both, in agreement with previous reports [21], [22]. The orbital fibroblast cells used in our experiments were all derived from orbital tissues of fat-predominant patients with minimally active GO. All seven GO patients were biochemically euthyroid with anti-hyperthyroid medications at the time of surgery, and their clinical activity scores were three or less. This could explain the similar responses of GO and normal cells to IL-1β, and this was the reason we used IL-1β to stimulate inflammation and hyaluronan production in orbital fibroblasts from both GO and normal donors. However, in naive normal cells without IL-1β stimulation, mRNAs of proinflammatory molecules were not detectable and not affected by quercetin pretreatment, whereas those molecules were expressed at detectable levels, and their expression was suppressed by quercetin, in GO cells. Although the GO patients in our study had minimal inflammatory activity by clinical criteria, the enlarged fatty tissue compressed under pressure in the fixed bony orbits of GO patients could be exposed to more inflammatory local conditions than normal orbital fatty tissues.

Quercetin significantly attenuated the IL-1β- or TNF-α-induced activity measured with an NF-κB-dependent reporter construct, and it also inhibited IL-1β-induced NF-κB nuclear translocation, suggesting that the anti-inflammatory action of quercetin may be mediated by the NF-κB pathway. In our study, an NF-κB inhibitor suppressed the IL-1β-induced ICAM-1 and COX-2 expression, but not that of IL-6 and IL-8. This indicates that different mechanisms are involved in the anti-inflammatory action of quercetin. We have also tested the effect of quercetin on the expression of IL-10, an anti-inflammatory cytokine repressing the inflammatory cytokines such as TNF-α, IL-6 and IL-1 by activated macrophage. The basal expression of IL-10 in GO cells was very weak, and was not also increased by IL-1β stimulation. Either stimulated with IL-1β or not, the quercetin did not affect the expression of IL-10 gene. The suppressive mechanism of quercetin on proinflammatory molecules may not be associated with IL-10. Further investigation is required to identify the signaling pathways involved in the action of quercetin on IL-6 and IL-8 expression in orbital fibroblasts.

GO is characterized by an inflammation of retrobulbar tissues, leading to accumulation of hydrophilic glycosaminoglycan, which in turn attracts water into surrounding tissues and thereby increases the volume of the orbital connective tissue and extraocular muscles [27], [28]. Orbital fibroblasts respond in vitro to various mediators of inflammation, including IL-1β, by producing excessive amounts of hyaluronan, a major glycosaminoglycan, in the orbital tissues of GO patients [28], [29]. A recent report showed that 75 µM quercetin treatment for 5 days reduces hyaluronan production; then when quercetin is removed, hyaluronan production increases at a similar trend, but at a lower level, to that observed in untreated fibroblasts [15]. It was also reported that proliferation of orbital fibroblasts is suppressed by continuous exposure to quercetin (75 µM for 3 days, or 30 µM for 5 days). Judging from the increased lactate dehydrogenase (LDH) reported to be released from these quercetin-treated cells, and the decreased proportion of cells in G2 and S phases, cell death or cell cycle blockade appears to be associated with quercetin treatment. In our study, the maximum quercetin treatment was for 24 h, and under this condition there was no evidence for either apoptotic or necrotic effects. Therefore the inhibitory effect of quercetin on proinflammatory molecules and hyaluronan that we observed is not associated with nonspecific drug cytotoxicity.

IL-1β and TNF-α are known to increase hyaluronan production [28], [29], and we found that the hyaluronan production stimulated by IL-1β or TNF-α was significantly inhibited by quercetin pretreatment for 24 h. This inhibition was not associated with suppression of the NF-κB pathway by quercetin, because NF-κB inhibitors did not reduce the level of hyaluronan (data not shown). Further studies are needed to identify the pathway by which quercetin inhibits hyaluronan production.

Quercetin was reported to affect adipocytes during specific stages of development, resulting in either inhibition of adipogenesis or induction of apoptosis [12], [30]. Quercetin inhibited lipid accumulation and induced apoptosis in early- and mid-phase maturing and lipid-filled mature primary human adipocytes [12]. Our microscopic results with Oil Red O staining show that quercetin suppresses adipogenesis in orbital fibroblasts and reduces the protein levels of adipogenesis-related transcriptional factors, PPARγ and C/EBPα, and their upstream regulator, C/EBPβ. The inhibitory effect of quercetin on adipogenesis is not associated with nonspecific drug cytotoxicity, as shown by MTT analysis of cell viability. It is known that the PPARγ and C/EBP transcription factors are expressed at distinct phases during adipogenesis, and they have been shown to play important roles: there is a positive feedback loop between PPARγ and C/EBPβ during the terminal stages of adipogenesis [31]. Our data suggest that quercetin exerted antiadipogenic effects by suppressing these adipogenic transcription factors. The potential of many natural products and various flavonoids, including genistein, docosahexaenoic acid, epigallocatechin gallate, quercetin, and resveratrol, to inhibit adipocyte differentiation and stimulate lipolysis in adipocytes has been reported [12]–[14], [30], [32]. These reagents could have a similar potential for drug development in GO.

Stimulation of orbital fibroblasts with IL-1β in vitro can mimic orbital inflammation in GO, and in our study, this cytokine promoted all three pathological aspects of GO: inflammation, hyaluronan production, and adipogenesis. Consistent with previous reports [16], [33], we found that IL-1β stimulated adipogenesis in orbital fibroblasts, and this may have important clinical implications. Quercetin not only suppressed IL-1β-induced proinflammatory molecule expression and hyaluronan production, but also inhibited adipocyte differentiation enhanced by IL-1β. Thus, IL-1β might present an attractive therapeutic target in GO. We investigated the effect of quercetin on TNF-α-induced upregulation of the same four proinflammatory molecules induced by IL-1β, but, interestingly, quercetin did not show inhibitory effects (data not shown).

Quercetin is now available in a high-grade purified form, and clinical phase I–III studies can be readily performed in the near future [34]. The beneficial effects of quercetin are supported by the detailed findings at the molecular and cellular levels of the specific pathways and molecules affected. However, many questions regarding flavonoids remain to be investigated. It is unknown whether they may contribute to the clinical benefits seen in the epidemiologic studies. However, we believe the results of our present study are noteworthy, and we propose that phytochemicals, such as quercetin, could be used as lead molecules to develop a new generation of drugs for the treatment of GO. Treatment with quercetin could be safer and have fewer side effects than high-dose glucocorticoids. Further research and clinical studies are necessary to ensure the safety of quercetin treatment and to ascertain the optimum doses for prevention and treatment of GO, bearing in mind that phytochemicals seem to have tissue- and concentration-specific effects.

## Materials and Methods

### Reagents

Quercetin (Q0125), Oil Red O, and the MTT assay kit were purchased from Sigma-Aldrich, Inc. (St. Louis, MO, USA). Dulbecco's modified Eagle's medium (DMEM), fetal bovine serum (FBS), penicillin, and gentamycin were purchased from Hyclone Laboratories, Inc. (Logan, UT, USA). Annexin V-FITC apoptosis detection kit was purchased from BD Biosciences (Franklin Lakes, NJ).A hyaluronan (hyaluronic acid) enzyme-linked immunosorbent assay (ELISA) kit was purchased from Echelon Biosciences (Salt Lake, UT, USA). Recombinant human IL-1β and TNF-α were purchased from R&D Systems (Minneapolis, MN, USA). Anti-PPARγ, anti-C/EBP α, anti-C/EBP β, and anti-β-actin antibodies were all obtained from Santa Cruz Biotechnology (Santa Cruz, CA, USA).

### Cell culture and differentiation protocol

Orbital adipose/connective tissue explants were obtained from seven GO individuals undergoing surgical decompression for severe proptosis associated with increased orbital fat volume, and tissue from seven control individuals with no history of GO or autoimmune thyroid disease was obtained in the course of orbital surgery for other noninflammatory problems (Table 1). The GO patients were not on steroid medication for at least 3 months before surgery and were euthyroid at the time of surgery. The orbital adipose tissue volumes were seriously enlarged in all GO patients. However, the clinical activity score at the time of harvest was below four in all patients (i.e., all the GO patients were not in an active inflammatory disease state). Orbital decompression surgery is usually not performed in the active disease, as surgery itself can aggravate inflammation and proptosis can recur postoperatively. None of the patients had been previously treated with orbital radiotherapy. The protocol for obtaining orbital adipose/connective tissue was approved by the Institutional Review Board of Severance Hospital, and written informed consent was obtained from all patients.

**Table 1:** Clinical characteristics of patients in the study.

Age (y)	Gender	Duration of GO (y)	CAS	Previous GO treatment	Proptosis R/L (mm)	Surgery performed
**GO patients:**						
53	F	2.2	3/7	GC	23/24	Decompression
46	F	0.8	2/7	GC	22/19	Decompression
41	F	2.3	1/7	GC	23/23	Decompression
55	F	1.4	0/7	None	20/23	Decompression
57	F	0.9	1/7	GC	22/24	Decompression
44	M	1.5	3/7	GC	25/25	Decompression
62	M	3	3/7	GC	24/19	Decompression
**Controls patients:**						
45	F	n/a	0	n/a	n/a	Orbital wall fracture
55	F	n/a	0	n/a	n/a	Orbital wall fracture
35	F	n/a	0	n/a	n/a	Orbital wall fracture
54	F	n/a	0	n/a	n/a	Orbital wall fracture
61	M	n/a	0	n/a	n/a	Evisceration
57	M	n/a	0	n/a	n/a	Evisceration
48	M	n/a	0	n/a	n/a	Evisceration

*Abbreviations*: CAS, clinical activity score; GC, glucocorticoids; n/a, not applicable; F, female; M, male; R/L, right or left eye.

Tissue explants were minced and placed directly in plastic culture dishes in DMEM containing 20% FBS, penicillin (100 U/mL), and gentamycin (20 µg/mL), allowing preadipocyte fibroblasts to proliferate. After fibroblasts had grown out from the explants, monolayers were passaged serially by gently treating with trypsin/EDTA, and cultures were maintained in 80-mm flasks containing DMEM with 10% FBS and antibiotics. Cell cultures were grown in a humidified 5% CO_2_ incubator at 37°C. The strains were stored in liquid N_2_ until needed, and they were used between the third and seventh passage.

After cells reached confluence in 6-well plates, differentiation of adipocytes was initiated by the following protocol. The culture medium were changed to serum-free DMEM supplemented with 33 µM biotin, 17 µM pantothenic acid, 10 µg/ml transferrin, 0.2 nM T3, 1 µM insulin (Boehringer-Mannheim, Mannheim, Germany), and 0.2 µM carbaprostaglandin (cPGI2; Calbiochem, La Jolla, CA, USA). For the first 4 days, 1 µM insulin, 1 µM dexamethasone, and 0.1 mM isobutylmethylxanthine were included in the media. The differentiation was continued for 10 days, during which the media was replaced every 3 days. A PPARγ agonist, rosiglitazone (10 µM, Cayman, Ann Arbor, MI, USA), was added from day 1 for further stimulation of adipogenesis.

### Cell viability and apoptosis assays

To evaluate the effect of quercetin on preadipocyte orbital fibroblast viability, orbital fibroblasts of normal and GO patients were seeded into 24-well culture plates (1×10^5^ cells/well) and treated with different concentrations of quercetin (10, 30, 50, or 100 µM) for 24 h. After treatment, cells were washed, incubated with 5 mg/ml MTT solution for 4 h at 37°C, then solubilized in ice-cold isopropanol and analyzed spectrophotometrically. Absorbance of the dye was measured at 560 nm, with background subtraction at 630 nm, with a microplate reader (EL 340 Biokinetics Reader; Bio-Tek Instruments, Winooski, VT, USA).

To evaluate the effect of quercetin on preadipocyte orbital fibroblast apoptosis, an annexin V/FITC kit was used to detect apoptotic cells. Cells were washed with isotonic phosphate-buffered saline (PBS) and then incubated in serum-free DMEM in the presence of different concentrations of quercetin for 6 or 24 h, after which the apoptosis assay was performed according to the procedure recommended by the manufacturer. For flow cytometric analysis, 1×10^4^ cells were excited at 488 nm, and emission was measured at 530 and 584 nm to assess FITC and propidium iodide fluorescence, respectively.

### Semiquantitative RT-PCR

Total RNA was extracted with TriZol (Invitrogen, Carlsbad, CA). cDNA was synthesized from 1 µg of total RNA in a reaction containing 2 µl of a 10 mM dNTP mixture, 0.5 µl of recombinant RNasin ribonuclease inhibitor, 1 µl of AMV reverse transcriptase (15 U), reverse transcription buffer, 1 µl of Oligo(dT)_15_ primer (0.5 µg; reagents from Promega Corporation, Madison, WI, USA). PCR was performed in a reaction containing 0.25 mM dNTP, 0.25 U Taq polymerase (iNtRON Biotechnology, Inc., Korea), 10 pmol primer pair, and 3 µl cDNA, in a thermal cycler (PerkinElmer, NY). PCR cycling conditions for amplification of GAPDH, IL-6, and IL-8 consisted of 30 cycles of three serial segments: 94°C for 30 s, 55°C for 1 min, and 72°C for 1 min; for amplification of ICAM-1, 34 cycles of: 94°C for 30 s, 65°C for 30 s, and 72°C for 1 min; COX-2 was amplified in 35 cycles of: 93°C for 30 s, 60°C for 30 s, and 72°C for 30 s; IL-10 was amplified in 35 cycles of: 94°C for 20 s, 61°C for 20 s, and 72°C for 20 s. Primers for ICAM-1 were 5′-GGC CTC AGC ACG TAC CTC TA-3′ (forward) and 5′-TGC TCC TTC CTC TTG GCT TA-3′ (reverse); for IL-6, 5′-TCA ATG AGG AGA CTT GCC TG-3′ (forward) and 5′-GAT GAG TTG TCA TGT CCT GC-3′ (reverse); for IL-8, 5′-TTG GCA GCC TTC CTG ATT TC-3′ (forward) and 5′-AAC TTC TCC ACA ACC CTC TG-3′ (reverse); and for COX-2, 5′-GTT CCA CCC GCA GTA CAG-3′ (forward) and 5′-GGA GCG GGA AGA ACT TGC-3′ (reverse); and for IL-10, 5′-CTG TGA AAA CAA GAG CAA GGC-3′ (forward) and 5′-GAA GCT TCT GTT GGC TCCC-3′ (reverse). GAPDH primers were 5′-GCC AAG GTC ATC CAT GAC AAC-3′ (forward) and 5′-GTC CAC CAC CCT GTT GCT GTA-3′ (reverse). Amplification bands were quantified by densitometry and normalized against corresponding GAPDH bands to control for PCR variability.

### Hyaluronan ELISA

Orbital preadipocyte fibroblasts were grown to confluence in 12-well plates and then incubated for indicated time periods with various concentrations of quercetin before stimulation with IL-1β or TNF-α. Supernatants from the cell cultures were collected, and hyaluronan concentrations were determined using a competitive binding hyaluronan ELISA kit according to the manufacturer's instructions. Absorbance of reactions was measured at 405 nm, and the percentage of binding was calculated for each sample. The concentration of hyaluronan in the sample was determined using a standard binding curve generated with known amounts of hyaluronan. Samples were diluted 1∶10 before analysis, and the average of triplicate assays was determined.

### Nuclear protein extraction

Orbital preadipocyte fibroblasts were plated in 100-mm dishes at 70% confluence and then incubated with various concentrations of quercetin for 24 h before stimulation with IL-1β for 16 h. Nuclear proteins for NF-κB western blot analysis were then isolated using a nuclear extraction kit (Cayman, Ann Arbor, MI) following the manufacturer's protocol. Briefly, cells were washed in 1 ml ice-cold PBS/phosphatase inhibitor solution, centrifuged at 300× *g* for 5 min, resuspended in 500 µl ice-cold hypotonic buffer, left on ice for 15 min, vortexed, and then centrifuged at 15,000× *g* for 30 s. Pelleted nuclei were gently resuspended in 50 µl of ice-cold nuclear extraction buffer, vortexed for 15 s and then placed on ice for 15 min; resuspension and incubation of nuclei were repeated for a total of 6 times and then the nuclear suspension was centrifuged at 15,000× *g* for 5 min at 4°C. Aliquots of the supernatant that contained soluble nuclear proteins were frozen in liquid nitrogen and stored at −70°C.

### Transfection and Luciferase Assays

Orbital fibroblasts were transfected using the LipofectAmin2000 reagent (Invitrogen). Empty vector control DNA was added to ensure that each transfection received the same amount of total DNA. To test the NF-κB-dependent transcriptional activity, NF-κB-luciferase plasmid was transfected into orbital fibroblasts. After 24 h, cells were pretreated with quercetin (0, 50, or 100 µM) for 24 h prior to stimulation with IL-1β (10 ng/ml) or TNF-α (10 ng/ml) for 16 h. Luciferase reporter assays were performed using a luciferase kit (Promega, Madison, WI) by following the manufacturer's protocol, and the luciferase reaction product was measured with a luminometer.

### Oil Red O staining of cells

Cells were stained with Oil Red O as described by Green and Kehinde [35]. A 0.5% (w/v) stock solution of Oil Red O in isopropanol was prepared. For the working solution, 6 ml of the stock solution was mixed with 4 ml distilled water, left for 1 h at room temperature, and then filtered through a 0.2-µm filter. Cells were washed twice with 1× PBS, fixed with 3.7% (w/v) formalin in PBS for 1 h at 4°C and stained with 300 µl Oil Red O working solution for 1 h at room temperature. The dishes were washed with distilled water before being visualized with an Axiovert (Carl Zeiss) light microscope and photographed at 40 and 400× magnification with an Olympus BX60 light microscope (Olympus, Melville, NY, USA).

To measure lipid accumulation, cell-bound Oil Red O was solubilized with 100% isopropanol, and the optical density of the solution was measured with a spectrophotometer at 490 nm. Experiments for the quantitative assessment of adipogenic differentiation were performed in triplicate in cells from different donors, and results were normalized to the absorbance of untreated control differentiated cells.

### Western blot assay

Differentiated cells were washed with ice-cold PBS and lysed on ice for 30 min in cell lysis buffer consisting of 20 mM HEPES (pH 7.2), 10% (ν/ν) glycerol, 10 mM Na_3_VO_4_, 50 mM NaF, 1 mM phenylmethylsulfonyl fluoride, 0.1 mM dithiothreitol, 1 µg/ml leupeptin, 1 µg/ml pepstatin, and 1% (ν/ν) Triton X-100. Reagents were obtained from Sigma-Aldrich (St. Louis, MO, USA). Lysates were centrifuged for 10 min at 12,000× *g*, and cell homogenate fractions were stored at −70°C before use. Protein concentrations in supernatant fractions were determined by the Bradford assay (BioRad). Equal amounts of protein (50 µg) were boiled in sample buffer and resolved by sodium dodecyl sulfate polyacrylamide gel electrophoresis in 10% (w/ν) gels. The separated proteins were transferred to polyvinylidene fluoride membranes (Immobilon; Millipore, Billerica, MA), probed overnight with primary antibodies in Tris Buffer Saline Tween 20, and washed three times with Tris Buffer Saline Tween 20. Immunoreactive bands were detected with horseradish peroxidase-conjugated secondary antibody, and the bound peroxidase was visualized using an enhanced chemiluminescence kit (Amersham Pharmacia Biotech) and exposure to X-ray film (Amersham Pharmacia Biotech). The relative amount of each immunoreactive band was quantified by densitometry and normalized to the β-actin level in the same sample.

### Statistical analysis

All experiments were performed at least three times, and samples were assayed in duplicate each time. For statistical analysis of semiquantitative PCR assays and western blots, the mean value and standard deviation (SD) was calculated for normalized measurements of each mRNA or protein from multiple (≥3) samples harvested from different individuals. Cumulative differences at various time intervals or concentrations of drugs were analyzed by the Friedmann test. The one-way analysis of variance test and Tukey's multiple-comparison test as a post-test were performed to achieve quantitative assessment of adipogenesis. Data between or within cell groups at different drug concentrations and incubation times were analyzed by the t-test or analysis of variance using the SPSS program for Windows, version 12.0.1 (SPSS, Chicago, IL, USA). A *P*-value of <0.05 or <0.001 (as specified for different experiments) was considered significant.

## Supporting Information

Figure S1 **Effect of quercetin on the expression of IL-10 mRNA in GO orbital fibroblasts.** (A) Orbital fibroblasts (5×10^5^) from GO patients pretreated with 0, 10, 30, 50, or 100 µM quercetin for 24 h were used to analyze for IL-10 expression by RT-PCR. (B) Cells pretreated with quercetin (0, 50 or 100 µM) for 24 hours were then stimulated with IL-1β (10 ng/ml) for 16 h, and were then used for RT-PCR analyses. The experiments were performed in triplicate with cells from three different donors, and the expression of IL-10 was similarly weak in all experiments.
(TIF)

Figure S2 **Examination of prestained orbital fibroblasts cultured in adipogenic medium under light microscopy.** Orbital fibroblasts from GO patients were differentiated in control adipogenic medium with no additions (A), supplemented with rosiglitazone (10 µM); (B), IL-1β (10 ng/ml); (C), or both rosiglitazone and IL-1β (D). Magnification was ×40, or ×400 (inset).
(TIF)
