# Supplementary figures and images for: Quercetin Inhibits IL-1β-Induced Inflammation, Hyaluronan Production and Adipogenesis in Orbital Fibroblasts from Graves' Orbitopathy
Source: PLoS One. 2011 Oct 19;6(10):e26261. doi: 10.1371/journal.pone.0026261 (PMC3198474; doi:10.1371/journal.pone.0026261)

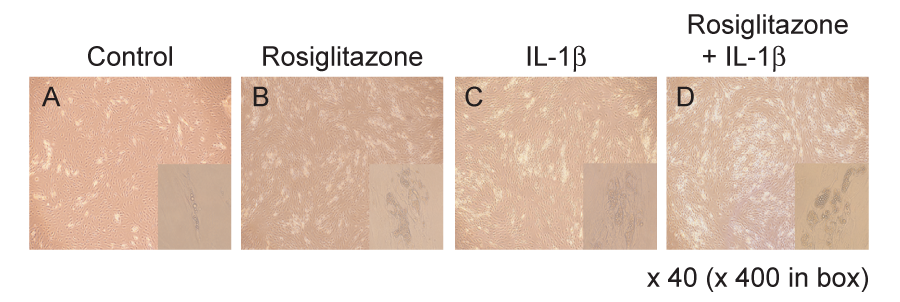

Supplement: Figure S2 — Examination of prestained orbital fibroblasts cultured in adipogenic medium under light microscopy. Orbital fibroblasts from GO patients were differentiated in control adipogenic medium with no additions (A), supplemented with rosiglitazone (10 µM); (B), IL-1β (10 ng/ml); (C), or both rosiglitazone and IL-1β (D). Magnification was ×40, or ×400 (inset). (TIF) [file pone.0026261.s002.tif]
